# AdPSO: Adaptive PSO-Based Task Scheduling Approach for Cloud Computing

**DOI:** 10.3390/s22030920

**Published:** 2022-01-25

**Authors:** Said Nabi, Masroor Ahmad, Muhammad Ibrahim, Habib Hamam

**Affiliations:** 1 Department of Computer Science, Virtual University of Pakistan, Rawalpindi 46300, Pakistan; said.nabi@vu.edu.pk; 2 Department of Computer Science, Capital University of Science & Technology (CUST), Islamabad 46300, Pakistan; masroor.ahmed@cust.edu.pk; 3 Department of Information Technology, University of Haripur, Haripur 22610, Pakistan; 4 Department of Computer Science and Statistics, Jeju National University, Jeju-si 63243, Korea; 5 Faculty of Engineering, Uni de Moncton, Moncton, NB E1A 3E9, Canada; habib.hamam@gmail.com; 6 Spectrum of Knowledge Production & Skills Development, Sfax 3027, Tunisia; 7 Department of Electrical and Electronic Engineering Science, School of Electrical Engineering, University of Johannesburg, Johannesburg 2006, South Africa

**Keywords:** meta-heuristic, PSO, inertia-weight, cloud, task scheduling, makespan, throughput

## Abstract

Cloud computing has emerged as the most favorable computing platform for researchers and industry. The load balanced task scheduling has emerged as an important and challenging research problem in the Cloud computing. Swarm intelligence-based meta-heuristic algorithms are considered more suitable for Cloud scheduling and load balancing. The optimization procedure of swarm intelligence-based meta-heuristics consists of two major components that are the local and global search. These algorithms find the best position through the local and global search. To achieve an optimized mapping strategy for tasks to the resources, a balance between local and global search plays an effective role. The inertia weight is an important control attribute to effectively adjust the local and global search process. There are many inertia weight strategies; however, the existing approaches still require fine-tuning to achieve optimum scheduling. The selection of a suitable inertia weight strategy is also an important factor. This paper contributed an adaptive Particle Swarm Optimisation (PSO) based task scheduling approach that reduces the task execution time, and increases throughput and Average Resource Utilization Ratio (ARUR). Moreover, an adaptive inertia weight strategy namely *Linearly Descending and Adaptive Inertia Weight (LDAIW)* is introduced. The proposed scheduling approach provides a better balance between local and global search leading to an optimized task scheduling. The performance of the proposed approach has been evaluated and compared against five renown PSO based inertia weight strategies concerning makespan and throughput. The experiments are then extended and compared the proposed approach against the other four renowned meta-heuristic scheduling approaches. Analysis of the simulated experimentation reveals that the proposed approach attained up to 10%, 12% and 60% improvement for makespan, throughput and ARUR respectively.

## 1. Introduction

Cloud Computing has revolutionized computing technology, where computing resources are accessed globally through the Internet [1]. These resources are provided in the form of services that can be easily and dynamically scaled-up and scaled-down by the Cloud users according to their needs [2,3]. The Cloud services are provided on a pay-as-go basis [4] to the users. The Cloud service model consists of Cloud service provider [5,6,7], Cloud user, and datacenter. A Cloud service provider acquires Cloud resources and provides these resources to the Cloud users according to their requirements. A Cloud data center represents the computing power of a Cloud which may contain hundreds of thousands of host machines. Each host on the data center may have one or more Virtual Machines (VMs) [8].

VM is considered an essential component of the Cloud environment. It enables the optimal use of the host machines in the Cloud computing environment [9]. VM provides flexibility to the Cloud operator to increase or decrease the number of CPUs, computation power of CPU, memory, and bandwidth according to its need. To select the most suitable resource among the resource pool for user’s jobs, task schedulers plays a key role. Tasks scheduling [4,7,10] is a principal component of the Cloud environment and the most challenging issue that needs to be optimized which is termed as an NP-hard problem [11].

Task scheduling approaches are categorized as heuristics, meta-heuristic algorithms, and hybrid of meta-heuristics and others approaches like heuristics and Machine learning among others. Heuristic-based algorithms provide near to optimal solutions for a specific problem. However, the meta-heuristics approaches are specifically designed for generalized optimal solutions that can be applied to multiple domains. Hybrid approaches are the combination of meta-heuristics, heuristics or machine learning based techniques for solving load-balanced task scheduling in Cloud computing [12,13,14]. This research focuses on meta-heuristics task scheduling algorithms.

The meta-heuristic based task scheduling schemes are divided into categories that are evolutionary-based like Genetic Algorithm (GA) [15], bio-meta-heuristics (swarm Intelligence-based), and non-bio-meta-heuristics like Simulation-Based Optimization (SBO) and Simulated Annealing (SA) [16]. Swarm Intelligence (SI) [17] is a sub-domain of computational intelligence and first used this concept by [18]. SI aims to solve computational problems by modeling self-organized populations of agents that can interact with each other. Agents can share their experiences by exchanging information. The interactions and movements of agents represent the population performance [19].

SI was first used by [18] for robotic intelligence in cellular robotic systems. After that, the definition of SI is expanded by [20] for algorithms and solving distributed problems. Currently SI is used for solving problems in various domains like health care, diagnosing diseases [21], stock analysis [22], academics [5], fraud and intrusion detection, feature selection [23], solving real-world engineering problems [24], pipe and road problems [25], data classification [26], Recommender Systems [12,13], and distributed computing and Cloud computing [27,28,29].

SI algorithms are broadly categorized into two sub-categories [30] i.e., (1) Sign based SI that is Bee Colony Optimization (BCO) and Ant Colony Optimization (ACO) [31], (2) Imitation based SI algorithms comprise of Cat Swarm Optimization (CSO) [32], Raven Roosting Optimization (RRO) [33], Improved RRO (IRRO) [30], Particle Swarm Optimization (PSO) [27,34,35], and Chicken Swarm optimization (CSO) [36] algorithms among others. The literature study shows that PSO is the most adopted optimization algorithm [19] for Cloud task scheduling.

Genetic Algorithm (GA) [15], SA [16], ACO [31], and PSO are the renowned meta-heuristic techniques use for cloud task scheduling. The meta-heuristic approach like ACO perform better at the early stage of optimization but converge slowly at later stage. PSO based schedulers perform better optimization than GA, has more natural computation background, fast convergence, and easy implementation as compared to GA.

One of the key factors in Cloud task scheduling is their computation time. Being a dynamic computation platform, Cloud schedulers should be fast and adoptable to the real Cloud platform. These scheduling schemes should have fast convergence and provide optimized solution. Therefore, this research focuses on optimizing tasks execution time, ARUR, and throughput [11] using PSO.

PSO can be applied to both discrete and continuous problems and is more efficient for global search in the problem space. PSO converges globally and tries to find a comparatively better fitness value. However, PSO is weak for local search and cannot pay more attention to the search in the local subspace. This increase the chances of trapping to the local optima which may have a lower convergence in the later stages. To overcome these limitations of PSO algorithms, inertia weights play a significant role. Inertia weight is an important control parameter for effectively adjusting the local and global search capability of the PSO scheduler. A small value of the inertia weight improves the local search while a bigger value of weight facilitates the global search. The literature study shows that there are five prominent and most commonly used inertia weights strategies out of a total of 15 inertia weights.

In this research, we contributed an adaptive meta-heuristic-based task scheduling approach which minimizes tasks execution time, enhances Cloud resource utilization, and throughput. The proposed approach favors the compute-intensive and independent Cloud tasks [37]. Moreover, a novel Inertia weight strategy i.e., *Leaner Descending and Adaptive Inertia Weight* (LDAIW) for PSO-based algorithms have been proposed. The proposed inertia weight strategy improves the performance of the PSO approach concerning the makespan [11], ARUR, and throughput. This is because the proposed technique provides a better integration of local and global search. The performance of the proposed approach has been investigated and compared against five inertia weight strategies. These weights are evaluated for optimizing the makespan, ARUR, and throughput in Cloud task scheduling. The major contribution of this research includes:

- In-depth study and critical analysis of state-of-the-art meta-heuristics based cloud task scheduling schemes to ascertain their application types, scheduling objectives, and limitations of these algorithms.
- Empirical evaluation of the most prominent and state-of-the-art inertia strategies for PSO-based algorithms.
- A adaptive PSO-based meta-heuristic task scheduling approach is proposed that reduces makespan, improves the resource utilization and throughput.
- A novel inertia weight strategy named *Leaner Descending and Adaptive Inertia Weight* (LDAIW) is designed and developed that improves the performance of PSO-based algorithms concerning makespan, throughput and ASRUR.
- A comparative experimental performance evaluation of AdPSO has been performed against their counterparts.

The rest of the paper is structured as follows: Section 2 presents the details concerning the related task scheduling work followed by Section 3 that delineates the details of the proposed approach. This section discuss the SWARM intelligence-based algorithms, PSO model, System model, Inertia weight strategy, proposed Inertia weight strategy, and proposed Task scheduler. The experimental configuration setup, dataset details and results and discussions are presented in Section 4. Section 5 discuss the conclusion and future work.

## 2. Related Work

The discussion concerning the state-of-the-art meta-heuristic task scheduling approaches is presented as follows. In [38] a hybrid load-balancing approach is proposed that combines Teaching Learning Based Optimization (TLBO) algorithms with the Grey Wolves Optimization (GWO) approach. The proposed approach combines the strengths of GWO and TLBO algorithms to effectively balance the load based on the time and related cost. This approach also reduces task waiting time in the tasks queue. However, throughput is not considered.

The authors in [39] have designed and developed a deadline aware task scheduling approach for Cloud Computing. The scheduling scheme has used the GA algorithm to enhance the execution time and cost of resources by considering variation in VM performance and acquisition delay. However, GA faces scalability issues for a large and complex problem.

Look-Ahead Genetic Algorithm (LAGA) is a modified form of the Genetic Algorithm (GA) that has been proposed by [1] for large-scale distributed systems such as Cloud and Grid computing environments. LAGA is considered suitable for run-time based scheduling of compute-intensive tasks and reliability. This approach identifies task orders based on the completion time of resources in every generation and chooses the resource that has a minimum failure rate during the mutation step. The reliability and task failure rate are the scheduling objectives of this approach. However, this approach does not consider makespan and throughput as scheduling criteria.

The authors have proposed a Node duplication-based Genetic Algorithm (NGA) is a GA-based algorithm designed for multi-processor heterogeneous systems [8]. The focus of NGA is on communication delay time and application completion time of the resources. The fitness function of this algorithm evolves in two steps. (1) Fitness of tasks that provided information regarding all other tasks to the system is scheduled for the legal order. Here the legal system schedules a pair of independent tasks on a single processor. (2) In this step, NGA looks at the processor fitness that tries to execute the task in minimum time. NGA inherits core issues of scalability for large and complex problems from the genetic algorithm.

The author in [40] has performed a comparison between the GA and PSO algorithms by using several test cases. It has been observed that in the majority of test cases, the PSO algorithm provides a better quality solution in a faster way than GA. Based on their experiments, it is claimed that for distributed systems the performance of the PSO algorithm is better than GA.

Author in [9] has proposed a task scheduling approach that combines Gravitational Emulation Local Search (GELS) and PSO. This approach aims to improve the makespan and tasks meeting their deadlines. However, throughput is not considered

In [41] authors have analyzed the PSO-based task scheduling algorithms in Cloud. The authors have classified the current PSO-based research work on the basis of the no of objectives that need to be optimized. This categorization of PSO algorithms includes a single objective or multiple objectives. To improve the solution quality, most of the researchers have applied basic PSO or updated PSO. The author has concluded that balanced scheduling and meeting Quality of Service (QoS) requirements (i.e., makespan, throughput, and resource utilization, etc.) required more focus and improvement. Inertia weight has not been considered for evaluation and analysis.

Authors in [30] has proposed IRRO and CSO based meta-heuristic algorithms. The proposed technique combines the strengths of CSO and IRRO algorithms that help in balancing the global and local search process. This approach has also proposed a dynamic scheduling framework named IRRO-CSO based Dynamic Scheduling Framework (ICDSF). Response time, premature convergence, makespan, and throughput have been used as evaluation parameters. However, ARUR is not considered as an evaluation parameter.

RTPSO-B a Rang and Tune based PSO with Bat technique has been proposed in [42]. RTPSO-B is an enhanced PSO-based algorithm that improves the efficiency of task scheduling in the Cloud. This algorithm solves the inertia weight issue of the existing PSO by introducing the data locality technique. The small inertia weight assists local search and the large inertia weight assists the global search process. For better optimization, the PSO approach has been combined with the Bat algorithm. Utilization of Cloud resources, makespan, cost are the key evaluation parameters however, throughput is not considered for evaluating scheduling algorithms.

Integer-PSO [43] is a discrete version of PSO based tasks scheduling algorithms in Cloud. Integer-PSO can be used for both single and multiple objectives optimization-based task scheduling in Cloud. The Integer-PSO has considered a bi-objective optimization problem. These objectives include task execution time and computation and management costs. However, the Integer-PSO does not consider throughput as a task scheduling objective and a fixed value is used for inertia weight.

In [44] authors have proposed a honey bee and improvement detection operator based load balancing algorithm named Hyper-heuristic. The proposed approach has the capability to distribute the cloud-based workload among the virtual machines with minimized makespan time. Hyper-heuristic scheduling scheme is evaluated against state-of-the-art in terms of makespan time, processing time, degree of imbalance. However, the Hyper-heuristic algorithm does not consider throughput and ARUR as scheduling objectives.

Authors have proposed a PSO based Task oriented Load Balancing (TBSLB-PSO) in [45]. TBSLB-PSO improves the load-balancing by migrating tasks from overload VMs to under-loaded VMs using the task migration technique. The proposed scheduling technique reduces the load balancing tasks by migrating tasks without stopping the overloaded VMs.

An adaptive Particle Swarm Optimization (APSO) based scheduling technique for Resource Constrained Project Scheduling Problems (RPSP) has been proposed in [46]. This technique aims to issue invalid particle generation. For this purpose, the authors have proposed a Valid Particle Generator (VPG) operator that is embedded with the PSO algorithm. The VPG convert the invalid particles to the valid one by in-degree and out-degree of activities in the directed acyclic graph. Moreover, the author has also proposed an adaptive inertia weight strategy using parameters like previous inertia weight, current iteration number, fitness value. Performance of the APSO is evaluated in terms of Makespan. However, throughput and ARUR are not used for evaluation.

Author in [28] has proposed a cloud task scheduling framework using a modified PSO (PSO-BOOST) based meta-heuristic algorithm. The proposed approach finds an optimized solution for conflicting objectives. This approach considers time, acceptance ratio, cost, and throughput as evaluation parameters. However, in this approach, the role and selection criteria of inertia weight have not been explicitly discussed. Moreover, a new compromise-optimized solution for conflicting metrics has been proposed using the principle of Pareto Optimal Theory (POT). ARUR is also not considered as an evaluation parameter.

Author in [24] has proposed an adaptive inertia weight approach based enhanced version of PSO. A set of ten (10) well-known test problems for optimization were used to evaluate and test their presented scheduling technique and four (4) other variants of PSO. The reason is that the performance of the PSO-based algorithms mostly depends on the selection of inertia weight strategy and optimal parameter setting. The proposed approach has also been evaluated for real-world engineering problems. This approach has been evaluated in terms of solution accuracy and convergence speed. Makespan and throughput are not considered as evaluation parameters.

Author in [47] has presented a review of different inertia weight strategies used by various researchers in their work. The author has classified these inertia weights into three groups includes time-varying, constant, and adaptive inertia weights strategies. The scheduling objective of this approach is the average makespan. However, throughput and ARUR are not considered.

Author in [48] has evaluated and compared five different inertia weights for the PSO algorithm. Makespan is considered as an objective function for the evaluation of inertia weights. The author has suggested that Linear Descending Inertia Weight (LDIW) performed better than other inertia weight strategies. However, throughput is not considered for evaluation. Table 1 presents a comparison of the existing task scheduling approaching highlighting the application type, strengths and weaknesses of each of the approach.

The in-depth investigation of the related literature shows that majority of the existing task scheduling approaches are evaluated using small datasets which is not enough to prove the scalability of these approaches. This is because scalability is an important factor in scheduling algorithms. Moreover, the inertia weight strategy is an important control parameter for PSO-based algorithms to balance the local and global search of particles. However, most of the existing scheduling techniques either used a constant value for Inertia weight or were not discussed explicitly. Similarly, the majority of the existing approach has not considered a makespan, ARUR, and throughput as scheduling objectives. To overcome these limitations, a novel and adaptive inertia weight strategy for PSO-based task scheduling algorithm has been proposed and compared with five most prominent inertia weight strategies and other PSO-based state-of-the-art task scheduling algorithms. The proposed approach uses four instances of a renowned HCSP based scientific benchmark dataset using makespan and throughput as scheduling objectives.

## 3. Proposed Approach

This Section delineates the methodology of our proposed task scheduling algorithm. The proposed methodology comprises the proposed inertia weight strategy and the proposed task scheduler. This section also describes the background knowledge of swarm based PSO algorithm.

### 3.1. PSO Model

PSO algorithm is a global search-based self-adaptive optimization approach [34]. This approach is population-based scheduling technique that relies on the social behavior of the particles. This is a swarm intelligence based approach that is inspired by the social behavior of the fish school and birds flock. The swarm population consists of generations and particles. Generations show the total number of iterations that need to be performed to get an optimized solution. Each generation has several particles and every particle in a generation shows a single solution. The number of generations and particles varies from case to case and is adjusted to get a more optimal solution. Every particle has a position, velocity, local/personal best (*pBest*), and global best (*gBest*). Personal best shows the most optimal solution of a particle while gBest shows the best solution among all particles. The velocity and position of all particles are updated in each iteration based on inertia weight, pBest, and gBest of the particle. Suppose D represents the dimension of solution space, where *X_i_* is a vector that represents positions of particles in a search space i.e.,

(1) $$X_{i}=\left(x_{i1},x_{i2},x_{i3},\ldots ,x_{id}\right)$$

In every iteration, particles constantly change their position and search for a more suitable solution.

(2) $$Pb_{i}=\left(pb_{i1},pb_{i2},pb_{i3},\ldots ,pb_{id}\right)$$

The *Pb*${}_{i}$ represents the best solution for each particle (as depicted in Equation (2)). V${}_{i}$ shows the velocity of particles(as shown in Equation (3)).

(3) $$V_{i}=\left(v_{i1},v_{i2},v_{i3},\ldots ,v_{id}\right)$$

Equation (4) is used by the PSO to the updated velocity of each particle

(4) $$v_{id}^{k+1}=w*v_{id}^{k}+c_{1}r_{1}\left(pb_{id}^{k}-x_{id}^{k}\right)+c_{2}r_{2}\left(Gb_{d}^{k}-x_{id}^{k}\right)$$

where *i* = 1, 2, 3,…, *n* (shows the no of particles), *k* = 1,2,3,…, *itrmax* (max. no of iterations that is 200 iterations in this article), D shows the number of dimensions or the no of tasks that need to be assigned to the VMs in an optimized manner. $x_{id}^{k}$ is the current position and $v_{id}^{k}$ is the current velocity of the $i^{\mathrm{th}}$ particle of $k^{\mathrm{th}}$ iteration in *d* dimensional space. The parameter *w* is the inertia weight that balances the global and local search of the particles, *c*_2_ and *c*_1_ are the constant acceleration factors. $r_{1}$ and $r_{2}$ are random values between 0 and 1. Equation (5) depicts the updated position of particles.

(5) $$x_{id}^{k+1}=\left(v_{id}^{k+1}+x_{id}^{k}\right)$$

The best value of every individual element (personal best) is computed by the fitness function which is based on maximization or minimization problem. The fittest element of all the individuals is termed as global best.

(6) FitnessFunction=Minimization(Objective)

The pBest value of each individual element is updated for each particle in all iterations if the new value is better than the current value. The PSO based heuristics computes and records the best value among all individuals (i.e., gBest shown in the Equation (7)) in the swarms.

(7) $$gBest=max(pBest_{1},pBest_{2},pBest_{3},\ldots ,pBest_{n})$$

To resolve the task scheduling issue using PSO-based heuristics and to enter a schedule as a search solution, identify suitable maps among PSO particles and problem solution [49]. Every particle of PSO indicates the possible solution for tasks to VM mapping. Table 2, Table 3 and Table 4 are used to illustrate mapping among PSO particles and problem solutions. Table 4 shows the tasks to VM mapping using PSO-based swarm intelligence. For this mapping, five VMs with different processing power (in Million Instruction Per Second (MIPS)) are used. VM${}_{1}$ has computation power of 50 MIPS, 100, 200, 350 and 500 MIPS that represents computation capability of VM${}_{2}$, VM${}_{3}$, VM${}_{4}$ and VM${}_{5}$ respectively (as shown in Table 2).

Table 3 shows 15 tasks with different computation requirements in term of Million Instructions (MIs) that need to be mapped on five VMs (Table 2).

Table 4 depicts the mapping of 15 tasks to 5 VMs, P11 (particle 1, iteration 1) represents the first possible solution, where Tsk${}_{5}$, Tsk${}_{10}$, Tsk${}_{15}$ are allocated to VM${}_{1}$; Tsk${}_{4}$, Tsk${}_{9}$, Tsk${}_{14}$ to VM${}_{2}$, Tsk${}_{6}$; Tsk${}_{13}$ to VM${}_{3}$, Tsk${}_{3}$, Tsk${}_{7}$, and Tsk${}_{12}$ to VM${}_{4}$; Tsk${}_{1}$, Tsk${}_{2}$, Tsk${}_{8}$, Tsk${}_{11}$ are assigned to VM${}_{5}$ respectively.

### 3.2. System Model

The objective of the task scheduling scheme is to choose the optimized task mapping strategy on the Cloud resources that can reduce the task execution time and improve ARUR, and the Cloud throughput. Considering user’s applications that consists of a set of D independent and compute-intensive tasks i.e., ${\mathrm{Tsk}}_{s}={\mathrm{Tsk}}_{1},{\mathrm{Tsk}}_{2},{\mathrm{Tsk}}_{3},\ldots ,{\mathrm{Tsk}}_{d}$ that need to be scheduled on a set of *M* VMs i.e., $T_{s}=T_{1},T_{2},T_{3},\ldots ,T_{d}$ that need to be scheduled on a set of *M* VMs, i.e., $\mathrm{VMs}=V_{1},V_{2},V_{3},\ldots ,V_{m}$ where D>>M. For instance, Table 2 shows VMs (*M* = 5) with their computation power in MIPS that needs to execute 15 tasks (i.e., D=15) with their computation requirements in MI. When task $T_{D}$ are mapped to ${\mathrm{VM}}_{j}$, the Expected Completion Time (ETC) of the assigned task is calculated using Equation (8):

(8) $${\mathrm{ETC}}_{dj}=RT_{j}+{\mathrm{EET}}_{dj}$$

where $RT_{j}$ is the ready time of ${\mathrm{VM}}_{j}$, i.e., the time needed for VM to complete already assigned workload. EET${}_{dj}$ is the Expected Execution Time (EET) of task Tsk${}_{D}$ on VM${}_{j}$ which is obtained by dividing task computation requirements (in MI) by computation power of VMs (in MIPS) and is described by formula given in Equation (9).

(9) $${\mathrm{EET}}_{Dj}=\frac{T{}_{D}\;\mathrm{size}}{{\mathrm{VM}}_{j}\;\mathrm{computation}\;\mathrm{power}}$$

Equation (10) computes the total time taken by VM${}_{j}$ to execute all of the assigned tasks denoted by CT${}_{j}$.

(10) $${\mathrm{CT}}_{j}=\sum_{D=1}^{S_{j}}\left({\mathrm{ETC}}_{Dj}\right)$$

where $S_{j}$ is the number of scheduled (assigned) tasks to ${\mathrm{VM}}_{j}$.

Makespan is the maximum completion time among all the VMs and is computed as shown in Equation (11). Minimized makespan shows better performance in terms of early execution of the workload.

(11) $$\mathrm{Makespan}=\max({\mathrm{CT}}_{1},{\mathrm{CT}}_{2},{\mathrm{CT}}_{3},\ldots ,{\mathrm{CT}}_{m})$$

where *m* is the number of VMs.

Throughput is another important evaluation metric in Cloud computing. Throughput is the ratio between the total number of tasks executed per unit time [11]. In our case, throughput of the whole Cloud datacenter is defined as the ratio between total number of tasks executed on a datacenter and makespan (shown in Equation (12)). Higher throughput represents better performance i.e., executing more tasks in a unit time.

(12) $$\mathrm{Throughput}=\frac{D}{\mathrm{Makespan}}$$

where *D* represents the total number of tasks executed in the Cloud data center.

The objective function of the proposed approach is based on the maximization problem and is computed using Equation (13).

(13) ObjectiveFunction=max(throughput+(1/makespan))

where makespan [11] represents the maximum execution time of the data center i.e., the execution time of VM that completes execution of the assigned task at the last of all other VMs and throughput in our case is the ratio between the total number of tasks executed by the Cloud and makespan. Higher throughput and reduced makespan give maximum value for our objective function.

### 3.3. Inertia Weight Strategies for PSO Model

The optimization procedure of all the swarm intelligence-based meta-heuristics consists of two major phases. These phases include local and global searches. The balance of global and local search has a key role in finding optimal solutions. For the ideal situation, at the start of the search procedure, the espousal of global search space should be more than a local search space [47]. It allows population-based meta-heuristics to explore more search space at the beginning and then finding the global optimal position with more care. Inertia weight is the strongest control factor to maintain local and global search in a balanced way [48].

The literature study show that various researchers have worked on the inertia weight selection strategy to balance the local and global search of particles. However, there is still need to improve this balance. Researchers have proposed several inertia weight strategies, however, some of these inertia weights are popular among the research community. These inertia weights strategies includes Simple Random Inertia Weight (SRIW) [50], Chaotic Inertia Weight (CIW) [47,51], Chaotic Random Inertia Weight (CRIW) [51], Linear Descending Inertia Weight (LDIW) [47,52], and Adaptive Inertia Weight (AIW) [53].

SRIW inertia weight was proposed by [50] represented by the formula (shown in Equation (14)).

(14) $$W_{\mathrm{SRIW}}=0.5+\left(0.5*rand\left(\right)\right)$$

where *rand*() represents random values between 0 and 1.

The formula (shown in Equation (15)) shows CRIW that is proposed in [51]

(15) $$W_{\mathrm{CRIW}}=\left(0.5*z\right)+\left(0.5*rand\left(\right)\right)$$

where *z* represents any value between 0 and 1, rand() represents random value between 0 and 1.

Equation (16) depicts the CDIW strategy that has proposed by [47,51,51]

(16) $$W_{\mathrm{CDIW}}=\left(w_{1}-w_{2}\right)*\left(\frac{MAXitr-Itr}{MAXitr}\right)+\left(w_{2}*z\right)$$

where $w_{2}$ and $w_{1}$ represents initial and final inertia weight and *z* is a random value between 0 and 1. MAXitr represents maximum iterations and Itr shows current iteration.

LDIW has been proposed by [47,52] which is represented by formula (depicted by Equation (17))

(17) $$W_{\mathrm{LDIW}}=\left(w_{1}-w_{2}\right)*\left(\frac{MAXitr-Itr}{MAXitr}\right)+\left(w_{2}\right)$$

where $w_{1}$ and $w_{2}$ are the initial and final values of inertia weight.

Author has proposed Adaptive Inertia Weight (AIW) strategies in [53] (shown in Equation (18)). AIW approach adjusts the weight value after each iteration using feedback received from the previous iteration. The feedback provides the success rate (i.e., $P_{s}$) of particles in their previous iterations. The success rate of the particle shows that how many times a particular particle improves local best values as compared to the previous one and the inertia weight is computed in Equation (18).

(18) $$W_{\mathrm{AIW}}=\left(\left(w_{1}-w_{2}\right)*P_{s}\right)+w_{2}$$

where $W_{\mathrm{AIW}}$ is the inertia weight calculated using AIW strategy, $w_{1}$ shows initial value, $w_{2}$ denotes the final value and $P_{s}$ is the particles success rate from previous iterations and is computed using Equation (20).

### 3.4. Proposed Inertia Weight Strategy

The performance of the PSO algorithm depends on optimal parameters setting and inertia weight. Searching for an optimal solution within the search region, the inertia weights bring a balance between exploration and exploitation [24]. In this paper, a novel inertia weight strategy “Linearly Decreasing Adaptive Inertia Weight (LDAIW)” has been proposed (shown in Equation (19)).

(19) $$W_{PA}=\left(\frac{\left(w_{1}-w_{2}\right)}{P_{s}}+\left(\frac{\left(MAXitr-Itr\right)}{MAXitr}\right)*\left(\frac{\left(w_{1}-\left(w_{1}-w_{2}\right)\right)}{P_{s}}\right)\right)$$

The proposed approach, exploits strengths of both LDIW [47,52] and AIW [53] strategies.

In LDIW, the weight value is set to the maximum in the start based on the assumption that global search is favored in the initial stage to explore more search space. The weight value of LDIW is decreasing gradually to decrease the search space gradually in local search at the end which leads to better performance than other state-of-the-art inertia weight strategies [48]. This is a widely used inertia weight strategy due to its simplicity and fast convergence; however, the state of the environment is not checked for adopting the inertia weight.

The AIW technique monitors the search space and adjusts weight value based on the feedback from one or more parameters. This method uses the success rate of the particles as a feedback parameter and adjusts the inertia weight strategy according to the state of the environment. It has been observed that AIW works well for smaller datasets as compared to larger one.

Based on these observations, a novel inertia weight strategy has been proposed that combines the strength of LDIW and AIW inertia weight strategies. The proposed strategy LDAIW has characteristic of linear decreasing behavior from LDIW (not in AIW) and adjust weight values based on feedback behavior from AIW (which not exist in LDIW). Being linearly decreasing and adaptive, LDAIW out perform in terms of Makespan, throughput, and ARUR than other state-of-art approaches.

In Equation (19), the value of $P_{s}$ is calculated using Equation (20). The value of $P_{s}$ can also approach to zero when there is no improvement in particle position (Pos) as given in Algorithm 1 line 3. If the value of $P_{s}$ becomes zero then it is assigned a default value of 1 as given in Algorithm 1 line no 35–37. In Equation (19), $w_{1}$ is the maximum value and $w_{2}$ is the minimum value. MAXitr represents the total number of iterations which is set to 200 in this research after fine-tuning, *Itr* shows the current iteration, and $P_{s}$ represents article success rate which is used as feedback for adjusting inertia weight as shown in Equation (20).

(20) $$P_{s}=\left(\frac{\sum_{i=1}^{n}\left(SS_{i}\right)}{N}\right)$$

Particle success rate ($P_{s}$) is computed using Equation (20), where N represents the total number of particles, and $SS_{i}$ is the success status of particles and is defined using Equation (21), and *n* represent index of particle i.e., *i* = 1 to *n*. The proposed weight strategy provides a better balance between global and local searches.

(21) $$SS_{i}=\left\{\begin{array}{cc} 1\; & \;pBest_{i}>pBest_{i-1}\\ 0\; & \;\mathrm{otherwise} \end{array}\right.$$

### 3.5. Proposed Scheduler

This section presents and discuss the proposed scheduling algorithm. A set of tasks with their computation requirements in Million Instructions (MI) and a set of VMs with their computation power in Million Instructions Per Second (MIPS) has been used as the input parameters. The output of the proposed approach includes the tasks to VM mapping.

At Line 1–2 (Algorithm 1), VM list (vmList) and task list (taskList) is obtained. The total number of tasks (taskCount) and total number VMs (vmCount) are computed (Line 3–4, Algorithm 1). Line 5-10 (Algorithm 1) presents the necessary initialization of the proposed approach. The initialization step of the particles has been performed and their results have been stored in pbMap (Line 11, Algorithm 1). The while loop (Line 12–39, Algorithm 1) executes according to a fixed number of times i.e., MaxItr which is 200 in our case. The Inertia weight has been computed (Line 13, Algorithm 1) based on Equations (19)–(21). The for loop (Line 14–37, Algorithm 1) repeats according to the number of particles which is 20 in this work. Each particle represents a complete mapping of tasks to VMs. The nested for loop (Line 15–28, Algorithm 1) iterates for each task to be mapped to the VM, where *r1* and *r2* are two random numbers between 0 and 1 (Line 17, Algorithm 1). The $c_{1}$ and $c_{2}$ are constant acceleration factors whose values are initialized at 2 and 1.49455 respectively [48].
**Algorithm 1:** Proposed PSO scheduler.
 **Input**: taskMap: Set of tasks with their length in MI and    vmMap: Set of VMs with their processing capacity(MIPS)
 **Output**: gbFMap: global best based final mapping of tasks to VMs vmList = getVmList(vmMap) taskList = gettaskList(taskMap) taskCount = taskList.size() vmCount= vmlist.size() pos = 0, v = Randnbr(0, 1), w = 0.0, SS = 0 noParticles = 20, itr = 1, MaxItr = 200 c${}_{1}$ = 2, c${}_{2}$ = 1.49455, w${}_{1}$ = 0.9, w${}_{2}$ = 0.4 pbMap<Integer, Double> = Null vRTMap <Integer, Double> = Null pMap<task, Vm> = Null prtsMap<Integer, Map<task, Vm>> = Null pbMap = initializeParticles(taskCount, vmCount, pbMap, pMap, gbFMap, noParticles) 
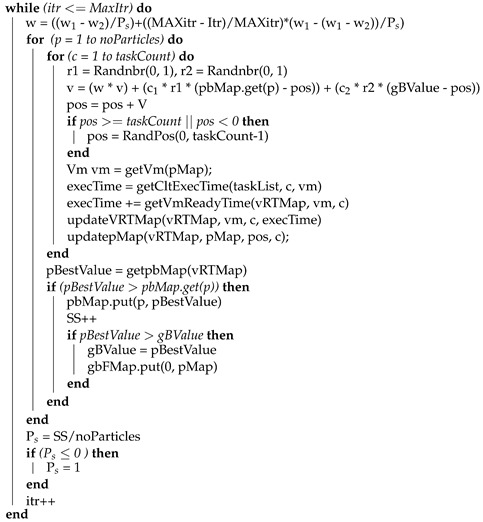



The velocity and position are updated (Line 18–19, Algorithm 1) using Equation (4) and (5) respectively. The if condition (Line 20–22, Algorithm 1) restricts the particle positions within the lower and upper bound of the search space. In case the particle position value exceeds the total count of tasks or less than zero (Line 20, Algorithm 1), a new random position is assigned to the particles (Line 21, Algorithm 1). The VM is identified in Line 23 of Algorithm 1 and the execution time of the task is computed (Line 24, Algorithm 1) using Equation (9). The completion time of the task is computed by adding the execution time and ready time of the VM (that is stored in vRTMap) to start task execution as represented in Equation (10) (Line 25, Algorithm 1). The VM ready time is updated in the vRTMap (Line 26, Algorithm 1) and particle is added to the map (Line 27, Algorithm 1). The Personal best value (pBestValue) is computed (Line 29, Algorithm 1) and current pBestValue is compared with the previous pBestValue (Line 30, Algorithm 1).

In case the current best value is greater than the previous one then the previous value is replaced with new pBestValue and stored in pbMap (Line 31, Algorithm 1). At line 32 (Algorithm 1), pBestValue is compared with the global best value (gBValue). When the if condition (Line 32, Algorithm 1) becomes true then gBValue is updated and mapping based on global best is updated in the global best based final mapping (gbFMap)(Line 33–34, Algorithm 1). This process continues until all tasks of single-particle are mapped to the VMs. In each iteration, the Itr is incremented by 1 (Line 38, Algorithm 1) which is used as a condition in the while loop at (Line 12, Algorithm 1).

## 4. Experimental Setup and Simulation Results

To evaluate and compare the proposed approach, we have implemented PSO algorithm with five different inertia weights proposed in [50,51,52,53]. These inertia weights strategies includes SRIW [50], CRIW [51], CIW [51], LDIW [52], and AIW [53]. The experiments simulate the optimized solution using makespan, ARUR, and throughput. The results of the proposed approach have been validated by comparing results generated using PSO with the other five prominent inertia weight strategies.

### 4.1. Dataset Analysis

Experiments have been performed using four different instances of HCSP benchmark dataset [11,54,55,56]. Based on our supposition that in real Cloud environment D>>M, each of the HCSP instances used has 8132 heterogeneous tasks and 16 heterogeneous VMs.Heterogeneous means that VMs in the VMs set are heterogeneous in terms of their processing capability (in MIPS) and tasks in the task set are heterogeneous in terms of tasks computation requirement (in MI). These instances include i_hilo, c_hilo, i_lohi, and c_lohi that represent different heterogeneity and consistency level in terms of VM computation capability (in MIPS) and tasks computation requirement (in Million Instructions(MI)) [54,55]. By consistent means, the variations in VMs MIPS and tasks MIs are uniform. However, inconsistent behavior shows that the variations in VM MIPS and tasks MI are not uniform. The c and i are used for the consistent and inconsistent nature of tasks and VMs respectively. Similarly, lo represents low and hi represents a high level of heterogeneity. The lohi shows a low level of heterogeneity for VMs and a high level of heterogeneity of tasks. High heterogeneity means that there are more variations in the sizes of tasks (i.e., there is a big difference between the smallest and largest task). Similarly, low heterogeneity represents a smaller difference between the largest and smallest tasks. The sizes of tasks in different HCSP instances are different like i.e., the sizes of tasks in i_hilo and c_hilo are smaller than that of i_lohi and c_lohi instances of the HCSP dataset and is considered as a bit lighter datasets than others.

### 4.2. Parameter Initialization

At the start of the search process, particles are initialized to some random positions as shown in the table Table 5. For the task to VM mapping, tasks are randomly assigned to VMs at this stage.

The maximum number of iterations and particles is fixed (200 iterations and 20 particles) after comprehensive fine-tuning by setting different values for maximum iterations and particle size. The lower bound and upper bound are set to zero and no of VMs-1 (VMCount-1) respectively. The values of w${}_{1}$ and w${}_{2}$ are set to 0.4 and 0.9, respectively [47,48]. The value of Acceleration factors c${}_{1}$ and c${}_{2}$ are initialized to 2 and 1.49455, respectively [48]. The stopping criteria are the maximum number of iterations (MaxItr), which equals 200.

### 4.3. Simulation Environment

This section presents the computational environment used for simulation. To perform experiments and evaluate the proposed scheduling technique, a simulation environment was used. This is because, in the simulation environment, any number and types of resources with various heterogeneity levels can be used for performing experiments. Moreover, experiments can be performed as many times as needed without any restriction of time and execution cost. The experimental setup for performing experiments consists of a PC equipped with a CPU (Intel core i5 T8500 3.0 GHz, Memory (20.00 GB)) HD 2 TB, implemented in Java-based Eclipse IDE 3.0 and Cloudsim 3.0.3 [11,57] simulation tool. Table 6 summarizes the experimental setup used for experimentation.

### 4.4. Experimental Execution and Results

Being stochastic optimization approaches, meta-heuristic-based algorithms need to be run multiple times to achieve meaningful and more realistic statistical evaluation [47]. In this research, every approach is executed 5 times for each instance of the HCSP dataset, and the average of these runs is computed and presented for the comparison.

**Figure 1:**
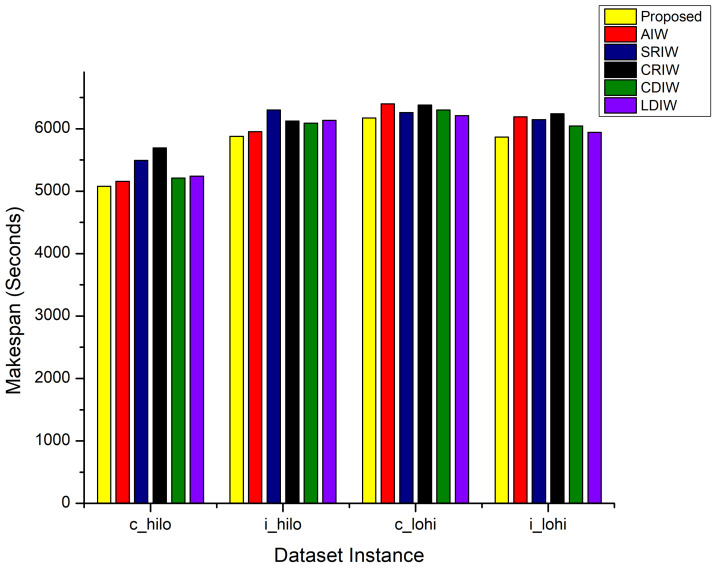
Makespan Comparison.

Makespan is the key performance evaluation parameter and ultimate demand of the cloud user. The results concerning the makespan for the AdPSO and available state-of-the-art approaches are plotted in Figure 1 for i_hilo, c_hilo, c_lohi, and i_lohi instances of the HCSP benchmark dataset. These results shows that SRIW, CRIW approaches has shown poor makespan performance for all the four instances of HCSP dataset. The CDIW, and LDIW approaches have shown improved behavior for c_hilo and i_hilo instances and slightly poor performance for c_lohi and i_lohi instances of HCSP dataset. The AIW approach has better performed for c_hilo and i_hilo instances as compared to c_lohi and i_lohi instances of HCSP dataset. This is because, the sizes of tasks in the c_hilo and i_hilo is smaller as compared to the tasks sizes in c_lohi and i_lohi HCSP instances. Moreover, LDIW strategy has shown consistent performance for all instance of the HCSP dataset due to its linearly decreasing mode. The AdPSO technique is capable to lower the makespan for all instances of HCSP dataset and improved by 1–7 % on i_hilo, 2–11% on c_hilo, 1–5% on i_lohi, and 1–4% on c_lohi instance of HCSP benchmark dataset as compared to AIW, SRIW, CRIW, CDIW, and LDIW state-of-the-art inertia weight strategies. This shows that the proposed approach maintain better balance between local and global search process.

**Figure 2:**
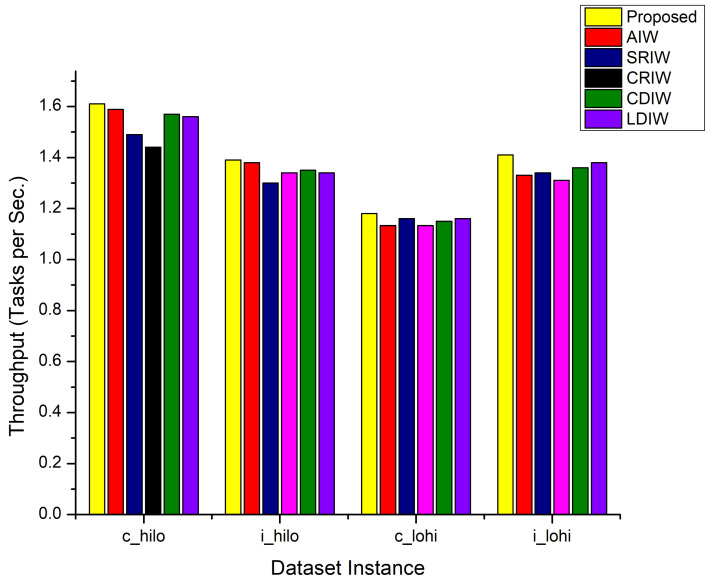
Throughput Comparison.

Another key parameter to compare the performance of the AdPSO is throughput. Figure 2 shows the throughput results for the AdPSO technique and compared contemporary approaches considering the c_hilo, i_hilo, c_lohi, and i_lohi instance of HCSP dataset. Likewise the makespan results, similar behavior is observed for the throughput results for all of the compared approaches. Figure 2 reveals that the proposed scheduler has achieved higher throughput up to 1–7% for the execution of c_hilo, 2–12% for i_hilo, 1–6% for c_lohi, and 1–4% for the execution of i_lohi instance of HCSP dataset as compared to PSO with AIW, SRIW, CRIW, CDIW, and LDIW inertia weight strategies.

**Figure 3:**
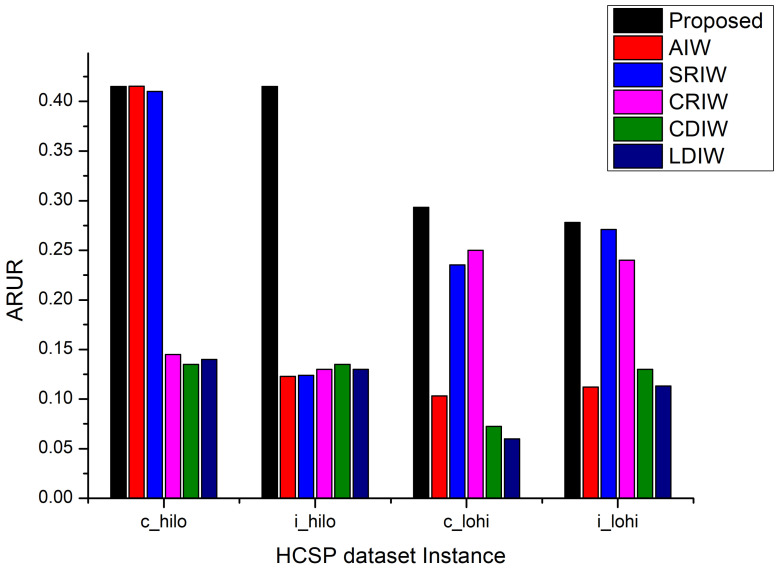
ARUR Comparison.

To perform evaluation of AdPSO, the ARUR results are obtained and compared against the AIW, SRIW, CRIW, CDIW, and LDIW approaches as shown in Figure 3. For the c_hilo instance, the AIW, SRIW and proposed approach have shown almost similar ARUR performance. The rest of the three approaches resulted in 60% less ARUR as compared to the AIW, SRIW, and proposed approaches. For the c_lohi and i_lohi datasets, the proposed approach dominated the rest of the contemporary approaches followed by SRIW and CRIW approaches. The AIW approach has shown poor ARUR performance for the i_lohi dataset instance whereas the LDIW approach has shown the lowest ARUR performance for the c_lohi dataset. However, for the i_hilo dataset, the proposed approach dominated all the approaches by almost 2 times against the other approaches.

The comparison results reveal that the proposed approach outperforms AIW, SRIW, CRIW, CDIW, and LDIW approaches for all four instances (i_hilo, c_hilo, i_lohi, and c_lohi) of the HCSP benchmark dataset. The reason is that the inertia weight strategy of the proposed approach is more effective in keeping a balance between local and global search.

**Figure 4:**
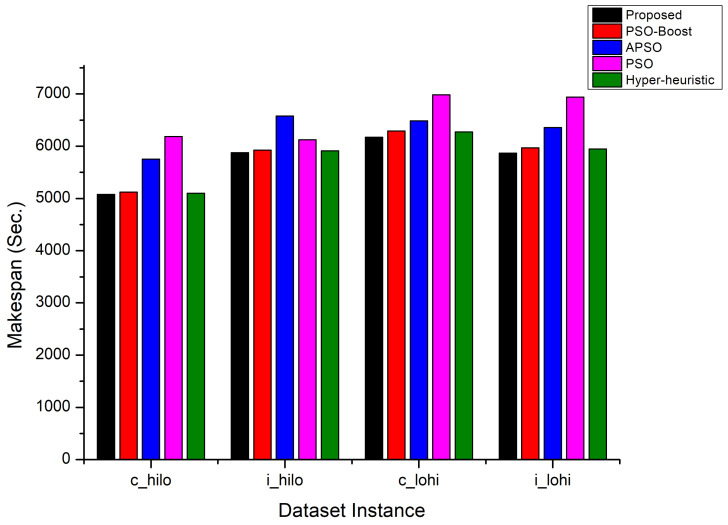
Makespan Comparison on HCSP dataset instances.

The evaluation is then extended and proposed PSO approach is compared against PSO-Boost, APSO, PSO, and Hyper-heuristic approaches using same HCSP benchmark datasets. The best and worst case results concerning the makespan for the compared approaches are shown in Figure 4. For the c_hilo dataset, the AdPSO has better performed the other three approaches by an improvement of 14–27%. The PSO-BOOST and Hyper-heuristic approaches have shown similar behavior for the i_hilo dataset instance while negligible degradation is observed for the c_lohi and i_lohi instances. The PSO approach has shown poor makespan performance whereas the APSO approach shown steady behavior. All these results advocates the effectiveness of the proposed approach.

**Figure 5:**
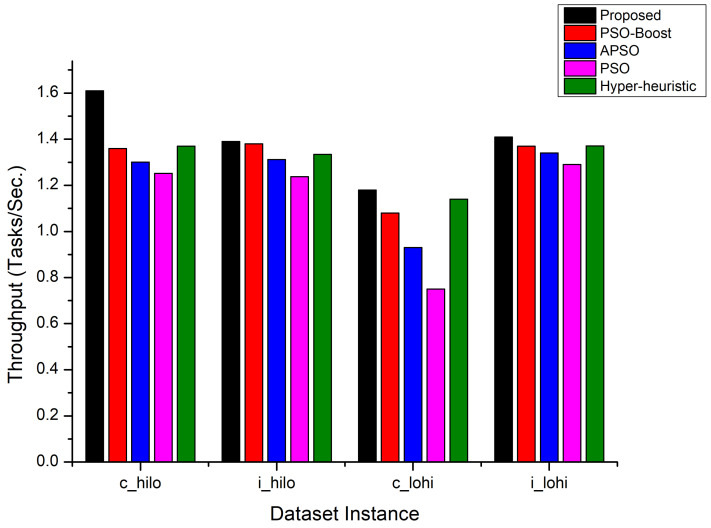
Throughput Comparison on HCSP dataset instances.

The results shown in Figure 5 show the throughput achieved for all the compared approaches. The empirical results (depicted in Figure 5) reveal that the proposed scheduling algorithm has improved the throughput by 6–22.32% against the compared approaches. Again the PSO-BOOST and Hyper-heuristic approaches attained second best throughput results and dominated the other compared approaches.

**Figure 6:**
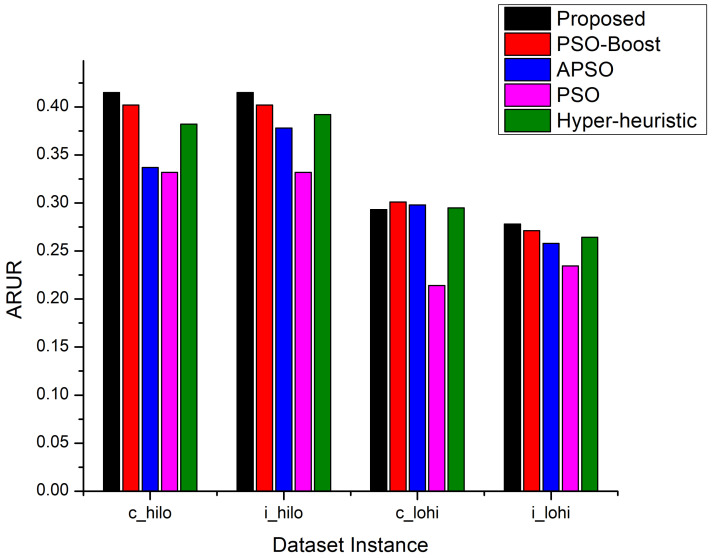
ARUR Comparison on HCSP dataset instances.

Another important metric to evaluate the efficiency of the task scheduling approaches is the Average Resource Utilization Ratio (ARUR). To evaluate the AdPSO technique against their counterparts, the experiments are performed using the same HCSP benchmark dataset instances. The experimental results shown in Figure 6 show that the proposed technique has gained 3.23%, 8.63%, 10.66% and 25% higher ARUR than PSO-Boost, APSO, Hyper-heuristic and PSO respectively for the c_hilo instance of the HCSP dataset. When the i_hilo dataset is used for the performance evaluation, almost same behavior is revealed for the approaches similar to that for c_hilo instance. similarly, the proposed approach attained 7% and 25% improved ARUR than APSO and PSO using c_lohi instance and 2.6%, 5.2%, 3.34% and 18.3% higher ARUR as compared to PSO-Boost, APSO, Hyper-heuristic and PSO for the i_lohi instance of HCSP benchmark dataset.

Experimental results show that AIW has acquired minimum overall execution time (makespan) and higher throughput as compared to SRIW, CRIW, CDIW, and LDIW for the execution of i_hilo and c_hilo instances of the HCSP dataset. This reveals that the performance of AIW is better in terms of makespan and throughput for comparatively smaller datasets than the larger one. This is because the size of i_hilo and c_hilo instances based workload are smaller in terms of total MI as compared to workload of i_lohi and c_lohi instances of HCSP dataset.

Experimental results show that the proposed approach has achieved 5.20 and 3.53% reduced makespan than AIW for i_lohi and c_lohi instances of the HCSP dataset respectively. These results also reveal that the proposed scheduling scheme has gained 5.35 and 4.09% higher throughput as compared to AIW for the execution of i_lohi and c_lohi instances of the HCSP dataset respectively. For the execution of i_hilo and c_hilo dataset, the proposed approach attained 1.58 and 1.25% reduced makespan and 1.59, 1.28% improved throughput as compared to AIW.

Our experimental results exhibit that LDIW results in reduced makespan and improved throughput as compared to AIW, SRIW, CRIW, and CDIW for the execution of i_lohi and c_lohi instances of HCSP benchmark datasets respectively. This shows that LDIW performs better in terms of makespan and throughput for larger datasets than smaller size datasets.

Experimental results show that the proposed scheduling scheme has achieved 4.19 and 3.15% reduced makespan than LDIW for the execution of i_hilo and c_hilo benchmark datasets respectively. These results also exhibit that the proposed scheduling approach has gained 4.34 and 3.28% improved throughput as compared to LDIW for i_hilo and c_hilo datasets respectively. Our experimental results show that the proposed approach achieved 1.27% minimized makespan than LDIW for the i_lohi dataset and 0.62% better makespan for the c_lohi dataset. These results show that the proposed approach has gained 1.38% higher throughput as compared to LDIW for i_lohi and 1.25% for c_lohi dataset.

The experimental results reveal that the proposed scheduling algorithm showed up to 26.84% lower makespan, 22.32% higher throughput, and 30% improved ARUR than PSO for the c_hilo, c_lohi, and i_lohi instances, respectively. Moreover, the proposed scheduling approach has achieved up to 20.12, 11.69, and 8.64% higher throughput as compared to the APSO algorithm. Similarly, the proposed scheduling algorithm has gained up to 14.97% lower makespan, 5.5% higher throughput, and 3.23% higher ARUR than the PSO-Boost scheduling algorithm. This is because of the proposed approach use of a novel inertia weight strategy to keep a balance between the local and global search. The proposed approach also exhibits stable performance for heterogeneous dataset. This is because the proposed approach inherent strengths of both AIW (better performance for smaller dataset) and LDIW (better performance for larger dataset) inertia weight strategies. The overall results reveal that the proposed approach outperforms concerning the makespan, throughput, and ARUR and is more stable and scalable than existing approaches. This research mainly focuses on the selection of inertia weight strategy to balance the global and local search using the makespan and throughput.

## 5. Conclusions and Future Work

PSO task scheduling approach is considered a more suitable approach for a load balanced scheduling of tasks due to its fast convergence and easy to implement nature. However, the PSO approach suffers from a pre-mature convergence issue. The inertia weight is a key attribute to keep a balance between global and local search space. In this paper, five inertia weight strategies have been investigated comprehensively using a PSO-based scheduler. This work contributed an adaptive inertia weight approach for Cloud-based task scheduling. The performance of the proposed approach has been evaluated and compared against five renown PSO based inertia weight strategies concerning makespan, throughput and ARUR. The results evaluation reveal that the proposed approach attained up to 10%, 12%, and 30% improvement concerning throughput and ARUR respectively against the compare approaches.

Most IoT applications [58,59] require real-time responses for accurate decision-making. As a future task it is intended to optimize the response time and employ scheduling to provide real-time or near to real-time response for delay-sensitive applications.

## Figures and Tables

**Table 1:** Summary of the related work.

Aproach	Application Type	Strengths	Weaknesses
GWO-TLBO [38]	11 benchmark functions	Consider Time and cost	Throughput not considered
GA [39]	Independent tasks	considering variation in VM performance and acquisition delay	Scalability issue and Throughput not considered
LAGA [1]	Independent tasks	Reduces the failure rate	Makespan and throughput not considered
NGA [8]	Workflow-based tasks	Support for communication delay and application completion time	scalability issue and Throughput not considered
GA vs PSO [40]	Test cases	Compared the performance of both PSO and GA	Makespan and throughput not considered
GELS-PSO [9]	10 well-known test problems	Improve makespan and maximize meeting task deadline	Throughput and ARUR not considered
PSO [41]	independent and workflow-based tasks	Consider both independent and workflow based workload for load balancing	Inertia weight strategy has not considered for analysis
ICDSF [30]	Independent tasks	Makespan, throughput and response time	ARUR not considered
RTPSO-B [42]	Independent tasks	ARUR, makespan, and cost	Throughput not considered
Integer-PSO [43]	Independent tasks	Support for makespan and cost	Throughput and ARUR is not considered and a constant value is used for inertia weight
PSO-BOOST [28]	independent tasks	considered throughput and conflicting parameters like makespan and cost	Role and selection criteria of inertia weight has not explicitly discussed, ARUR not considered
AIWPSO [24]	10 set of benchmark problems	Accuracy and convergence speed	Makespan and throughput not considered
PSO [47]	Workflow based tasks	Average makespan	Throughput and ARUR not considered
MIPSO [48]	Independent tasks	Makespan	Throughput and ARUR not considered

**Table 2:** Computation power of VMs.

VMs	VM${}_{1}$	VM${}_{2}$	VM${}_{3}$	VM${}_{4}$	VM${}_{5}$
VMs Computation power(in MIPs)	50	100	200	350	500

**Table 3:** Computation requirements of Tasks.

Task	Tsk${}_{1}$	Tsk${}_{2}$	Tsk${}_{3}$	Tsk${}_{4}$	Tsk${}_{5}$	Tsk${}_{6}$	${\mathrm{Tsk}}_{7}$	Tsk${}_{8}$	Tsk${}_{9}$	Tsk${}_{10}$	Tsk${}_{11}$	Tsk${}_{12}$	Tsk${}_{13}$	Tsk${}_{14}$	Tsk${}_{15}$
MIs	50	100	150	200	300	450	500	600	700	900	1200	1500	2000	3000	4000

**Table 4:** Tasks to VM mapping.

Task	Tsk${}_{1}$	Tsk${}_{2}$	Tsk${}_{3}$	Tsk${}_{4}$	Tsk${}_{5}$	Tsk${}_{6}$	Tsk${}_{7}$	Tsk${}_{8}$	Tsk${}_{9}$	Tsk${}_{10}$	Tsk${}_{11}$	Tsk${}_{12}$	Tsk${}_{13}$	Tsk${}_{14}$	Tsk${}_{15}$
P11	VM${}_{5}$	VM${}_{5}$	VM${}_{4}$	VM${}_{2}$	VM${}_{1}$	VM${}_{3}$	VM${}_{4}$	VM${}_{5}$	VM${}_{2}$	VM${}_{1}$	VM${}_{5}$	VM${}_{4}$	VM${}_{3}$	VM${}_{2}$	VM${}_{1}$
P12	VM${}_{1}$	VM${}_{5}$	VM${}_{3}$	VM${}_{2}$	VM${}_{1}$	VM${}_{5}$	VM${}_{3}$	VM${}_{4}$	VM${}_{4}$	VM${}_{5}$	VM${}_{1}$	VM${}_{3}$	VM${}_{2}$	VM${}_{5}$	VM${}_{4}$
P13	VM${}_{2}$	VM${}_{1}$	VM${}_{3}$	VM${}_{3}$	VM${}_{5}$	VM${}_{4}$	VM${}_{5}$	VM${}_{3}$	VM${}_{1}$	VM${}_{4}$	VM${}_{2}$	VM${}_{5}$	VM${}_{4}$	VM${}_{2}$	VM${}_{3}$
P14	VM${}_{3}$	VM${}_{2}$	VM${}_{5}$	VM${}_{4}$	VM${}_{4}$	VM${}_{1}$	VM${}_{1}$	VM${}_{5}$	VM${}_{2}$	VM${}_{3}$	VM${}_{5}$	VM${}_{1}$	VM${}_{2}$	VM${}_{4}$	VM${}_{2}$
—	—	—	—	—	—	—	—	—	—	—	—	—	—	—	—
P44	VM${}_{2}$	VM${}_{4}$	VM${}_{5}$	VM${}_{1}$	VM${}_{3}$	VM${}_{1}$	VM${}_{2}$	VM${}_{4}$	VM${}_{5}$	VM${}_{2}$	VM${}_{3}$	VM${}_{1}$	VM${}_{4}$	VM${}_{3}$	VM${}_{5}$

**Table 5:** Initialization parameters.

Parameters	Values
Total${}_{\mathrm{iterations}}$	200
Total${}_{\mathrm{particles}}$	20
Min${}_{\mathrm{position}}$	0
Max${}_{\mathrm{position}}$	No of VMs-1
w${}_{1}$	0.4 [47,48]
w${}_{2}$	0.9 [28,47,48]
Acceleration${}_{\mathrm{factor}}$ c${}_{1}$	2 [48]
Acceleration${}_{\mathrm{factor}}$ c${}_{2}$	1.49455 [48]
Stopping Criteria	MaxItr

**Table 6:** Summary of simulation environment configuration.

Parameters	Values
Simulator	Cloudsim version 3.0.3
processor	Intel cor i5-8500 3.00 GHz
RAM	20 GB
Hard drive	2 TB
Total host machines	10
Host machines Power	15,000 MBs each
VMs	16
Total tasks	8132

## Data Availability

Not required as dataset is provided in the manuscript.
